# Nanofibrous scaffolds for the guidance of stem cell-derived neurons for auditory nerve regeneration

**DOI:** 10.1371/journal.pone.0180427

**Published:** 2017-07-03

**Authors:** Sandra Hackelberg, Samuel J. Tuck, Long He, Arjun Rastogi, Christina White, Liqian Liu, Diane M. Prieskorn, Ryan J. Miller, Che Chan, Benjamin R. Loomis, Joseph M. Corey, Josef M. Miller, R. Keith Duncan

**Affiliations:** 1 Kresge Hearing Research Institute, Department of Otolaryngology-Head & Neck Surgery, University of Michigan, Ann Arbor, MI, United States of America; 2 Geriatrics Research, Education, and Clinical Center (GRECC), VA Ann Arbor Healthcare Center (VAAAHC), Ann Arbor, MI, United States of America; 3 Department of Biomedical Engineering, University of Michigan, Ann Arbor, MI, United States of America; 4 Departments of Otorhinolaryngology, Guangzhou First Peoples' Hospital and First Affiliated Hospital, School of Medicine, Jinan University, Guangdong, China; 5 Department of Materials Science and Engineering, University of Michigan, Ann Arbor, MI, United States of America; 6 Department of Neurology, University of Michigan, Ann Arbor, MI, United States of America; University of Pennsylvania, UNITED STATES

## Abstract

Impairment of spiral ganglion neurons (SGNs) of the auditory nerve is a major cause for hearing loss occurring independently or in addition to sensory hair cell damage. Unfortunately, mammalian SGNs lack the potential for autonomous regeneration. Stem cell based therapy is a promising approach for auditory nerve regeneration, but proper integration of exogenous cells into the auditory circuit remains a fundamental challenge. Here, we present novel nanofibrous scaffolds designed to guide the integration of human stem cell-derived neurons in the internal auditory meatus (IAM), the foramen allowing passage of the spiral ganglion to the auditory brainstem. Human embryonic stem cells (hESC) were differentiated into neural precursor cells (NPCs) and seeded onto aligned nanofiber mats. The NPCs terminally differentiated into glutamatergic neurons with high efficiency, and neurite projections aligned with nanofibers *in vitro*. Scaffolds were assembled by seeding GFP-labeled NPCs on nanofibers integrated in a polymer sheath. Biocompatibility and functionality of the NPC-seeded scaffolds were evaluated *in vivo* in deafened guinea pigs (*Cavia porcellus*). To this end, we established an ouabain-based deafening procedure that depleted an average 72% of SGNs from apex to base of the cochleae and caused profound hearing loss. Further, we developed a surgical procedure to implant seeded scaffolds directly into the guinea pig IAM. No evidence of an inflammatory response was observed, but post-surgery tissue repair appeared to be facilitated by infiltrating Schwann cells. While NPC survival was found to be poor, both subjects implanted with NPC-seeded and cell-free control scaffolds showed partial recovery of electrically-evoked auditory brainstem thresholds. Thus, while future studies must address cell survival, nanofibrous scaffolds pose a promising strategy for auditory nerve regeneration.

## Introduction

Disabling hearing loss affects approximately 360 million individuals worldwide [1]. The most common cause is sensorineural hearing loss, which is based on impairment of cochlear sensory hair cells and/or neurons of the auditory nerve [2,3]. Auditory sensorineural impairment, results from—among others—noise exposure, aging, traumatic brain injury, ototoxic drugs, disease and inherited disorders [2,4]. Remarkably, recent findings suggest that the causative role of auditory neuropathy and its prevalence are highly underestimated due to the insufficiency of standard auditory exams to detect neuronal deficits and research focus on sensory hair cells [5–8]. Thus, neuropathy is a likely basis for deficits in speech recognition and other auditory processing tasks in the absence of a permanent threshold shift measured in quiet [6]. Furthermore, when both the auditory nerve and cochlear hair cells are impacted, neural impairment can constitute a functional bottleneck and limit the success of interventions targeting the auditory periphery, including cochlear implants [3,9,10].

Neuron loss leads to permanent deficits due to the inability of mammalian auditory SGNs to spontaneously regenerate. Hence, multiple strategies have been advanced to either halt or reverse damage or replenish SGNs. These include local neurotrophin [3,9,11–15] and drug treatment [10,16,17] to promote recovery and re-sprouting of dendrites [18], enhancement of support from Schwann cells [19], in situ differentiation of progenitor cells [20,21], transdifferentiation [22] and stem cell therapy [23–25]. In cases of SGN degeneration, stem cell therapy poses several advantages. Use of stem cells would enable replacement without modification of host tissue. Moreover, stem cells could be more easily tailored to mimic adult SGN physiology and modified to preferentially target specific cell types in the cochlea and brainstem. With progress in the field of induced pluripotent stem cells, it is also becoming feasible to induce autologous transplants that combine the benefits of stem cells and avoidance of a deleterious host immune response.

The realization of successful nerve regeneration faces a number of challenges. One that is common for most, if not all, of the strategies discussed above is the recapitulation of native topology. SGNs are bipolar neurons that convey information from the sensory hair cells to the cochlear nucleus without any intermediate connections [26]. Replaced SGNs, whether from exogenous neural progenitors or endogenous transdifferentiation, must properly integrate into the auditory circuit making functional connections with their target cells. Yet, few attempts have been made to guide cells towards the native auditory innervation pattern [27]. Thus far, most therapeutic approaches have relied on self-organization of injected cells for obtaining a desirable phenotype, morphology, and innervation of target cells [23,25,28–33]. The unique environment in the adult compared to the developing nervous system will undoubtedly require further tissue engineering to optimize integration. The spatiotemporal distribution of morphogens and guidance cues as well as the formation of pathfinding tracts are essential to normal circuit development but change substantially during maturation [34]. Guidance is therefore a fundamental feature of cell integration that must be addressed in stem cell therapy.

Nanofibers bear the potential to provide guidance for neurite outgrowth and promote the establishment of proper connectivity. This approach takes advantage of cellular sensitivity to three-dimensional environments and extracellular contact cues [35–38]. In this regard, topographical cues not only promote directionality but induce intracellular signaling to affect differentiation and fate [39]. Besides guiding alignment [38,40–43], our labs and others have shown that nanofibers can enhance neuronal differentiation and neurite outgrowth, affect proliferation, and promote viability [38,42,44–49]. Further, chemical and electrical modification of nanofiber substrates can harness cell contact signaling and electrical activity to provide additional support for proper integration and survival [50]. Moreover, the design of a neuralized nerve prosthesis can be adjusted to optimize the integration of neurons with cochlear implants and provide a more comprehensive therapy for hearing loss [51]. To address the challenge of proper transplant cell pathfinding in the auditory system, we have designed a novel cell-seeded, nanofibrous scaffold for implantation in live, deafened guinea pigs.

## Materials and methods

### Stem cell maintenance and differentiation

Human embryonic stem cell lines used in this study were obtained from WiCell Research Institute, including the unmodified H7 cell line (WA07) and another line constitutively expressing hrGFP (H9 Cre-LoxP, referred to throughout as H9-GFP). Stem cells were maintained under feeder-free culture conditions using mTeSR1 media (STEMCELL Technologies) and passaged onto stem cell-qualified Matrigel coated substrates using dispase (2 mg/ml in DMEM/F12). Passages were limited to 20 from a starting passage number of P31 for WA07 and P22 for H9-GFP.

A stepwise neurodifferentiation procedure was used, modified from Kim et al. [52]. Briefly, the hESCs were isolated with trypsin and gently spun into AggreWell 800 plates to form embryoid bodies (EBs) in NEB media (DMEM/F12, 1x N2, 2x B27, 55 μM β-mercaptoethanol, 50 ng/ml noggin, and 500 nM dorsomorphin) supplemented with 10 μM Rho-kinase inhibitor (Y-27632, Millipore). About 10,000 cells were distributed into each microwell. Aggregates were cultured for 2 days in NEB media at 37°C in a 5% CO_2_, humidified incubator. The EBs were harvested from the microwells, passed through a 40 μm-mesh reversible cell strainer (Corning) to remove single cells and small clumps of cells, and cultured in NEB media in ultra-low-attachment plates for an additional day. To generate neural rosettes, the EBs were subsequently transferred to Matrigel-coated tissue culture plates and cultured in neural induction media containing NEB media supplemented with 0.5 μM purmorphamine and 0.5 μM all-trans retinoic acid (RA). The rosettes formed over the next 5 to 8 days, while exchanging the media every other day. When rosettes were clearly visible, 20 ng/ml FGF2 was added to the media for an additional day before harvesting.

Rosettes were collected manually then dissociated as NPCs using trypsin-EDTA to be frozen for later use, expanded in culture, or terminally differentiated. For expansion, NPCs were grown on Matrigel-coated culture plates at a density of 1–2 x 10^6^ cells/cm^2^ using neural induction media with 20 ng/ml FGF2. On the first day of each passage, the media was supplemented with 10 μM Y-27632. Media was fully exchanged every other day during expansion. NPCs were passaged up to 3 times to limit uncontrolled growth of contaminating non-neural “flat” cells. Neurodifferentiation was induced by plating cells at a density of 1–3 x 10^4^ cells/cm^2^ on Matrigel-coated substrates in terminal differentiation media (TD: Neurobasal, 1x N2, 2x B27, 1x GlutaMax, 1x non-essential amino acids, 55 μM β-mercaptoethanol, and 1 μM dibutyrl-cAMP). TD media was fully changed every other day.

### Quantitative PCR

Total RNA was extracted from samples of hESCs, EBs, rosettes, and NPCs using RNeasy Mini Kit (Qiagen). RNA quality was tested by Agilent 2100 BioAnalyzer with all samples exhibiting RIN scores of 8.0 or higher. First-strand synthesis was produced using SuperScript III reverse transcriptase (Invitrogen), and quantitative PCR (qPCR) reactions performed on an Applied Biosystems StepOne Plus thermocycler using Universal PCR Master Mix (Applied Biosystems) and Taqman qPCR Probes (Applied Biosystems). Reactions were performed in triplicate and cycle-thresholds averaged if the standard deviation for the triplicates was under 1.0. Fold change was calculated using the ΔΔCt method, normalizing each sample to the geometric mean of the housekeeping genes *GAPDH* (HS02758991_g1) and *RPS16* (HS01598516_g1) and calculating fold change relative to results from hESCs. Tested probes included *ASCL1* (Hs04187546_g1), *POU4F1 (BRN3A)* (Hs00366711_m1), *GATA3* (Hs00231122_m1), *NES* (Hs04187831_g1), *NEUROD1* (Hs01922995_g1), *NEUROG1* (Hs01029249_s1), *POU5F1 (OCT4)* (Hs04260367_gH), *PAX2* (Hs01057416_m1), *PAX6* (Hs00240871_m1), and *PAX8* (Hs01015257_g1).

### Quantification of neurite alignment on nanofiber mats

NPCs were terminally differentiated on Matrigel coverslips and aligned and unaligned two-dimensional nanofiber mats to determine impact under long-term growth conditions. Plasma treated polycaprolactone (PCL) nanofiber mats were obtained from Nanofiber Solutions. Fiber mats were coated with Matrigel and seeded at a density of 2 x 10^4^ in TD media with media changes every 3 days. To visualize neurite alignment and assess phenotype, preparations were immunostained with TUJ1 primary antibody as described below. Epifluorescence images were obtained with a BX51WI Olympus microscope with Orca Flash4.0 V2 Digital CMOS camera. Images were analyzed by fast Fourier transform (FFT) as described elsewhere [53], averaging intensities in a radial band 20–40 μm from the image origin and plotting against corresponding angle from the origin in 1° increments. From this plot, the full width-half maximum (FWHM) was calculated as a measure of strength of alignment.

### Nanofiber scaffold construction

An implantable scaffold was constructed of a nanofiber bundle inside a stiff polymer sheath. The custom-made polymer sheath consisted of a hollow PCL tube 1.7–1.95 mm in length, approximately 0.7 mm in outer diameter, and about 0.2 mm thick. In brief, the PCL sheaths were made by coating a 27G needle with 25% (w/v) PCL dissolved in chloroform. This needle was rotated at a velocity of 100 RPM to facilitate smooth coating and was repetitively dipped into the PCL solution using a linear stage (10 sec coating every 90 sec). After 10 min of coating, the PCL-coated needle was allowed to dry for 15 min. After completely drying, excess polymer was cut from the needle tip and fine forceps were used to remove the newly formed hollow PCL tube from the needle.

Nanofibers for the scaffolds were produced by electrospinning a 4:1 blend of PLLA and PCL dissolved in a 9:1 mixture of chloroform and dimethylformamide. The solution was delivered through a blunt-tip needle using a syringe pump advancing at 0.3 ml/hr. The tip of the needle protruded through the center of a 10 cm x 10 cm aluminum sheet charged to 20 kV. The rotating disc collector was placed 30 cm away, was spun at a velocity of 800 rpm, and contained a counter-charge of -2 kV. Nanofibers were collected until a desired density was obtained and then cut free of the rotating disc. Low-pressure vacuum was used to pull nanofiber bundles through the hollow PCL sheath. The ends of the fiber bundle were adhered to a coverslip and the device plasma oxygen treated for 3 min to increase hydrophilicity. Within 24 hours of plasma treatment, the sheath with nanofiber bundle (referred to throughout as the “scaffold”) was coated with Matrigel overnight at 4°C. After coating, coverslips and fiber bundles were seeded with a suspension of NPCs (20 μl of 10,000 cells/μl) and incubated at 37°C for 1 hour to promote cell adhesion. In some instances, these NPCs were pre-labeled with a 1:250 dilution of FluoroRuby (D1817, Life Technology, 5% stock concentration in water) in TD media for 24 hours before seeding the nanofiber bundles. Following the 1 hour incubation, the sheath was moved down the fiber bundle to cover an exposed, cell-seeded section of the bundle and trimmed at sheath openings to produce an implantable cell-seeded scaffold (NPC scaffold). These scaffolds were cultured in TD media for up to 24 hours before implantation.

### Animals

Guinea pigs were chosen because of the anatomical advantages in size of the IAM and well-established surgical approaches to the cochlear duct. Hartley guinea pigs of both genders approximately 2.5–3.5 weeks old (301–350 g) were obtained from Charles River Laboratories. Guinea pigs were housed in a temperature-controlled room on a 12:12h light:dark cycle. Animals were fed Certified Guinea Pig Diet #5025 (PMI Nutrition International, LLC) *ad libitum* and were provided with standard enrichment.

All subjects received analgesics (ketoprofen 1 mg/kg subcutaneously) prior to and for at least 48 hours after all surgeries. Antibiotics (Sulfatrim Pediatric Suspension) were given orally before and after implantations. Animals were anesthetized with xylazine (10 mg/kg) and ketamine (40 mg/kg) intramuscularly for deafening procedures and auditory assessments. A xylazine reversal agent (yohimbine, 1 mg/kg subcutaneously) was administered to facilitate recovery. Isoflurane gas anesthesia was administered via a mask for implantation procedures. Lidocaine (4 mg/kg subcutaneously) was infiltrated along incision lines prior to surgery. Body temperature was maintained on water-circulating heating pads or radiant heat panels. Supportive care with subcutaneous saline and an herbivore supplement was given as needed after all anesthetic procedures. Only left ears were manipulated

This study was conducted in accordance with the *Guide for the Care and Use of Laboratory Animals of the National Institutes of Health*. All experimental procedures were approved by the University of Michigan Institutional Animal Care and Use Committee (Protocol Number: PRO00006573).

### Deafening procedure

Subjects were deafened by application of ouabain into scala tympani of the inner ear and on the round window membrane using aseptic technique. A classic post-auricular approach was used to expose the cochlea. The middle ears were inspected carefully to ensure no sign of infection. When necessary, a diamond burr was used to remove small protrusions of bone above the round window to enlarge the niche. A microcannula was used to inject 5 μl of 10 mM ouabain (Sigma) through the round window. Gelfoam® moistened with an additional 5 μl of ouabain was placed on top of the round window membrane so that it expanded to fill the round window niche. The bulla defect was sealed with carboxylate cement followed by a 2-layer subcuticular and skin closure.

### Auditory brainstem response measurements

Auditory brainstem responses from acoustic (aABR) and electrical (eABR) stimuli were recorded essentially as described elsewhere [54]. Briefly, these experiments were conducted in an electrically and acoustically shielded chamber using Tucker Davis Technologies (TDT) System II hardware and SigGen/BioSig software (TDT) to present the stimulus and record responses. Animal subjects were anesthetized, and body temperature was maintained on a water circulating heating pad. Ear canals were examined via a stereoscope to insure an unobstructed ear canal, intact tympanic membrane, and a normal appearing middle ear. Acoustic click stimuli were 100 μs in duration and delivered at 5 pulses/s via a tube connected to a transducer (Beyer B4-31.05–00 headphone element; Beyer Dynamic) coupled to the external auditory meatus. The click stimuli were calibrated with a Brüel & Kjær 1/4” condenser microphone. Needle electrodes were inserted into the subcutaneous tissue at the vertex of the head (active) and the ventrolateral regions below the pinnae of the right (ground) and left ears (reference) for measurement of the aABR. Neural responses from up to 1024 sweeps were amplified (10,000x), bandpass filtered (0.3 to 3 kHz) and averaged using SigGen/BioSig software. Threshold was determined by proceeding from high to low intensities in 5 dB steps until the lowest stimulus intensity evoking a replicable waveform above noise was identified. Baseline aABRs were obtained approximately 1 week prior to deafening and repeated 2 weeks post-deafening to determine the ouabain-induced threshold shift. The post-deafening assessment included masking of the contralateral ear with 70 dB SPL white noise to prevent a crossover response from the contralateral-hearing ear. Subjects with a threshold shift less than 60 dB were eliminated from the study. For eABR measurements, epidural recording screws were used to collect the neurologic response to 50 μs computer-generated monophasic current pulses presented at 50 pulses/s with an alternating polarity on each presentation. Up to 2048 neural responses were amplified (10,000x), bandpass filtered (0.1 Hz to 10 kHz) and averaged for each intensity measured. As in the aABR procedures, threshold was determined by proceeding from high to low stimulus intensities using 10 μA steps near threshold to identify the lowest stimulus intensity producing a replicable waveform above noise levels. The eABRs were collected 9, 19, and 28–30 days after cell and electrode implantation.

### Implantation of NPCs

Two weeks after deafening, NPCs were introduced into the guinea pig IAM via cell-seeded nanofiber scaffolds or by cell-only injection. Epidural recording screws were placed on the skull [54]. The carboxylate cement from the deafening procedure was removed to expose the middle ear, which was then examined for abnormalities. The antereoventral portion of the bulla defect was drilled to improve the angle of approach to the IAM. A hole was drilled with a diamond burr in the basal turn of the cochlea about 0.5 mm inferior to the round window niche and 0.1 mm anterior to the ridge of the basal turn near the supporting wall.

For the cell injections, the fine tip of a custom-made microcannula [55] was forward-filled with 5 μl of NPCs (10,000 cells/μl), leaving a small air gap between the NPC media and saline-filled cannula. The cannula was attached to a Hamilton syringe with a 30G needle on a preprimed syringe pump. The cannula was then inserted into the IAM to a depth of 1mm, until a silastic ball placed on the cannula covered the cochleostomy defect. The cannula was temporarily secured at the bulla defect with a small drop of Vet-Bond tissue adhesive. The syringe pump was used to deliver the NPCs at approximately 1 μl/min.

For scaffold implantations, a diamond burr was used to expose the opening to the IAM. Three experimental groups were examined: sham, cell-free scaffolds, and NPC scaffolds. Sham controls included a brief implantation of the PCL conduit sheath into the IAM to induce acute damage introduced by the conduit alone. In these controls, the conduit was immediately removed and the animal allowed to recover. Cell-free and NPC scaffolds were permanently implanted into the IAM and monitored briefly to ensure stability within the internal canal. To electrically stimulate neural structures in the region, a custom platinum-iridium ball electrode, approximately 450 μM in diameter, was inserted into the cochleostomy and placed lateral to the end of the scaffold. The electrode wire was secured to the bulla defect and a piece of fascia was placed over the cochleostomy to limit leakage of CSF and movement of the electrode and implant. The electrode ground terminated in the bulla against the temporal bone. A percutaneous connector was secured to the dorsal skull with methyl methacrylate. The bulla defect was covered with carboxylate cement, further securing the electrode. The subcuticular layer and skin were closed.

### Histological processing of temporal bones

For plastic sections, animals were transcardially perfused with 2% glutaraldehyde in 0.15 M cacodylate buffer. The cochleae were extracted and fixed further in this same solution for 2 hours followed by decalcification in 5% EDTA (0.4% PFA) for up to 3 weeks. The specimen were dehydrated in increasing concentrations of ethanol and embedded in JB-4 resin. Thin, mid-modiolar sections (3 μm thick) were obtained from ouabain-deafened and control specimen. Sections were stained with toluidine blue and every third to sixth section imaged to limit double-counting surviving SGNs. The number of intact neural cell somas were counted in each Rosenthal’s canal and expressed as cell density by normalizing the counts to canal cross-sectional area. The average cell density was determined for 6 sections per specimen and then averaged across treatment group.

For cryosections, animals were transcardially perfused with 4% paraformaldehyde (PFA). Cochleae were quickly removed, fixed for an additional 2 hours in 4% PFA, and decalcified in 5–10% EDTA (0.4% PFA) for 2–3 weeks. After decalcification, the cochleae were cryopreserved in increasing concentrations of sucrose from 5% to 30% over 1–2 hours followed by an overnight incubation in 30% sucrose at 4°C. Subsequently, the cochlea were infused with increasing concentrations of Tissue-Tek O.C.T. embedding media in 30% sucrose for 1–2 hours followed by an overnight incubation in 100% O.C.T. at 4°C. Cochleae were frozen on dry ice. Serial sections (10 μm thick) were obtained through the cochlea along a mid-modiolar axis and/or radially throughout the entire length of the IAM. Sections were collected in a staggered manner such that each slide contained 8 to 12 sections from regions throughout the IAM.

### Immunohistochemistry

Cell preparations were fixed in 4% PFA for 10 min on coverslips and fiber mats or 60 min on nanofiber bundles. After fixation, these cell preparations and cryosections were processed similarly for immunohistochemistry. All specimens were blocked with DPBS+ (Dulbecco's phosphate-buffered saline supplemented with 5% normal donkey serum and 0.1% Triton X-100) for 15–60 min followed by overnight incubation at 4°C with primary antibodies in DPBS+. On the following day, preparations were washed extensively and stained with AlexaFluor-conjugated secondary antibodies (Invitrogen) for 1–2 hours followed by 5 min exposure to Hoechst 33342 (1–10 μg/ml, Invitrogen) to label nuclei. The specimens were imaged with epifluorescence using an Olympus BX51WI microscope outfitted with an Orca Flash4.0 V2 digital CMOS camera under the control of MetaMorph imaging software. Confocal images were taken with an Olympus FV10i.

### Antibodies

Primary antibodies included rabbit anti-hrGFP (Agilent Technologies, 240242, 1:200–1000), mouse anti-tubulin beta-3 chain (TUJ1 mouse, Covance/BioLegend, MMS-435P, 1:300), rabbit anti-tubulin beta-3 chain (TUJ1 rabbit, Covance/BioLegend, MRB-435P, 1:300 or Sigma T2200, 1:200), mouse anti-nestin (Millipore, MAB5326), rabbit anti-VGLUT1 (Synaptic Systems, 135 303, 4 μg/ml), rabbit anti-MAP-2 (Millipore, AB5622, 1:500), mouse anti-synaptophysin (SYP, BD Biosciences, 611880, 1:200), rabbit anti-CD45 (AbdSerotech, MCA1130 1:250), rat anti-CD11b (integrin alpha-M, BioRad, MCA74GA, 1:100), mouse anti-L1cam/MAC387 (BioRad, MCA874GT, 1:100), mouse anti-CD4 (BioRad, MCA749S, 1:100), rabbit anti-vimentin (Abcam, AB92547, 1:2000), and mouse anti-GFAP (Sigma, G3893, 1:2000).

### Statistical analyses

Statistical tests were implemented using SYSTAT 12.0. Comparisons between two means were accomplished with a Student’s t-test, while comparisons among several groups were examined with one-way or two-way analysis of variance (ANOVA) with post-hoc pairwise comparisons (Tukey HSD) when required. Statistical differences in qPCR data were tested by one-way ANOVA using ΔCt values for individual probes. In general, the standard significance level was set to 0.05.

## Results

**Fig 1:**
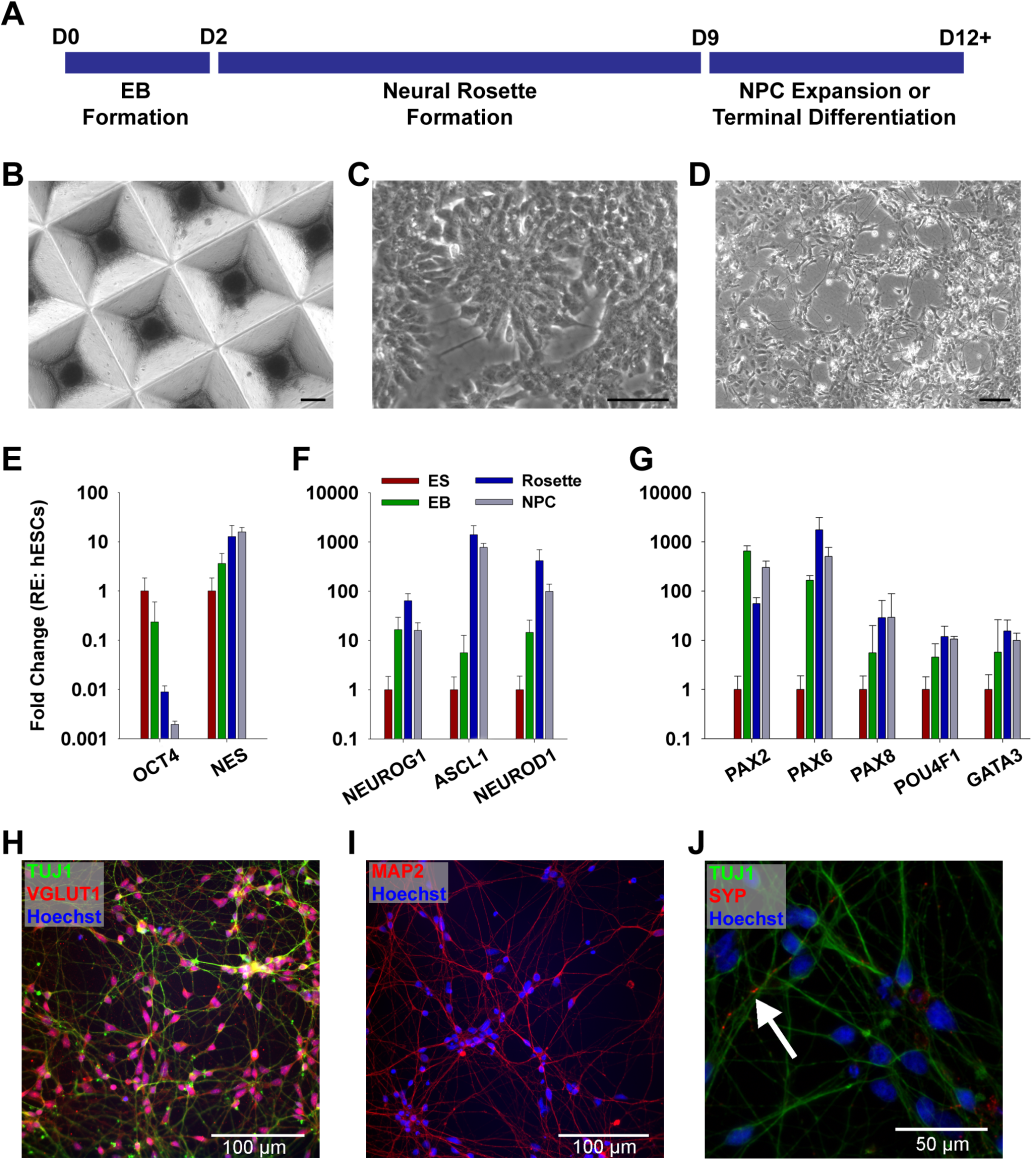
Neuronal differentiation protocol and phenotype analysis. (A) Illustration of the neuronal differentiation protocol. (B–D) Representative images of H7 hESC derived precursors after formation of EBs (B), neuronal rosettes (C) and NPCs (D). (E–G) qPCR analysis of gene expression during EB (green, n = 3), neuronal rosette (blue, n = 4) and NPC (grey, n = 3) stages compared to undifferentiated hESC (red, n = 3). Error bars indicate standard error of the mean. (H–J) Representative images of protein expression 4 weeks after terminal differentiation. Nuclei are counterstained with Hoechst. (H) Differentiated cells express the neuronal marker TUJ1 and the vesicular glutamate transporter VGLUT1. (I) MAP-2 expression indicates maturation of derived neurons. (J) Neurons stained positive for the synaptic protein synaptophysin (SYP, counterstained with TUJ1). Scale bars represent 200 μm in B, 50 μm C and J, and 100 μm in D, H, and I.

For differentiation of hESC to NPCs for implantation, we adapted a small molecule approach previously shown to produce mature, electrically active, glutamatergic neurons from hESC and hiPSC within 4 to 6 weeks [56]. The stepwise protocol generated NPCs within 12 days (Fig 1A), using spin-aggregation in micropatterned substrates to produce embryoid bodies (EBs, Fig 1B) followed by the formation of neural rosettes in adherent culture (Fig 1C) and ultimately the production of isolated NPCs (Fig 1D).

PCR analysis evidenced significant loss of the pluripotency marker *POU5F1* (*OCT4*) and upregulation of the neuroectoderm marker *nestin* (*NES*) at Rosette (n = 4; *OCT4 p* < 0.001, *NES p* = 0.025) and NPC stages (n = 3, *OCT4 p* < 0.001, *NES* p = 0.013) compared with undifferentiated hESCs (n = 3; Fig 1E). To test if differentiation was consistent with a fate comparable to SGNs, we probed for expression of *Neurogenin-1* (*NEUROG1*), a pro-neural transcription factor required for SGN development [57] (Fig 1F). *NEUROG1* was upregulated over hESCs at the completion of all 3 differentiation stages (EB *p* = 0.03, Rosette *p* = 0.0014, NPC *p* = 0.018). *NEUROG1* downstream target *NEUROD1* [57] and the pro-neural transcription factor *ASCL1* [58] were upregulated in EBs, Rosettes and NPCs (*NEUROD1*: EB *p* = 0.036, Rosette *p* < 0.001, NPC *p* = 0.003) and Rosettes and NPCs (ASCL1: Rosette *p* < 0.001, NPC *p* < 0.001), respectively. Also, the expression of *NEUROD1* and *ASCL1* in Rosettes and NPCs was enhanced compared to EBs (NEUROD1: Rosette *p* = 0.0078, NPC *p* = 0.046; *ASCL1*: Rosette *p* = 0.0013, NPC *p* = 0.0041).

In an extended analysis of otic developmental marker genes, we also found expression of the otic placode markers *PAX2* and *PAX8* [59], as well as the SGN markers *POU4F1 (BRN3A)* [60] and *GATA3* [61–63] (all *p* < 0.05 for EB, Rosette and NPC vs hESC; Fig 1G). However, we also found expression of *PAX6*, a gene frequently involved in neuronal differentiation as well as anterior adenohypophyseal, olfactory and lens placodes [64]. Kim et al. [56] reported that the addition of the sonic hedgehog (*SHH*) signaling pathway activator purmorphamine after the formation of EBs was able to suppress *PAX6* expression. Thus, we added purmorphamine and retinoic acid during the formation of rosettes, as both pathways are critical for ventral patterning and otic development [65–67]. While we observed some expression changes with the addition of both substances and decided to keep them in the protocol, we did not observe a substantial *PAX6* suppression compared to initial cultures without their addition (data not shown).

Subjecting NPCs to terminal differentiation shows that the derived neurons indeed adopt a glutamatergic fate (Fig 1H), as determined by expression of the vesicular glutamate transporter 1 (*SLC17A7*, also known as VGLUT1), which is also expressed by SGNs [68]. However, we also found some co-expression of ChAT (data not shown), suggesting glutamatergic-cholinergic co-transmission, which has been reported in the forebrain [69] and is consistent with a protocol aiming at forebrain neurons [56]. We did not find evidence for an inhibitory phenotype; specimens were negative for VGAT (vesicular inhibitory amino acid transporter, data not shown). Further, neurons expressed the maturation marker MAP-2 and the synaptic protein synaptophysin (Fig 1I and 1J). Thus, overall, the derived neurons showed a desirable phenotype for implantation into the inner ear.

**Fig 2:**
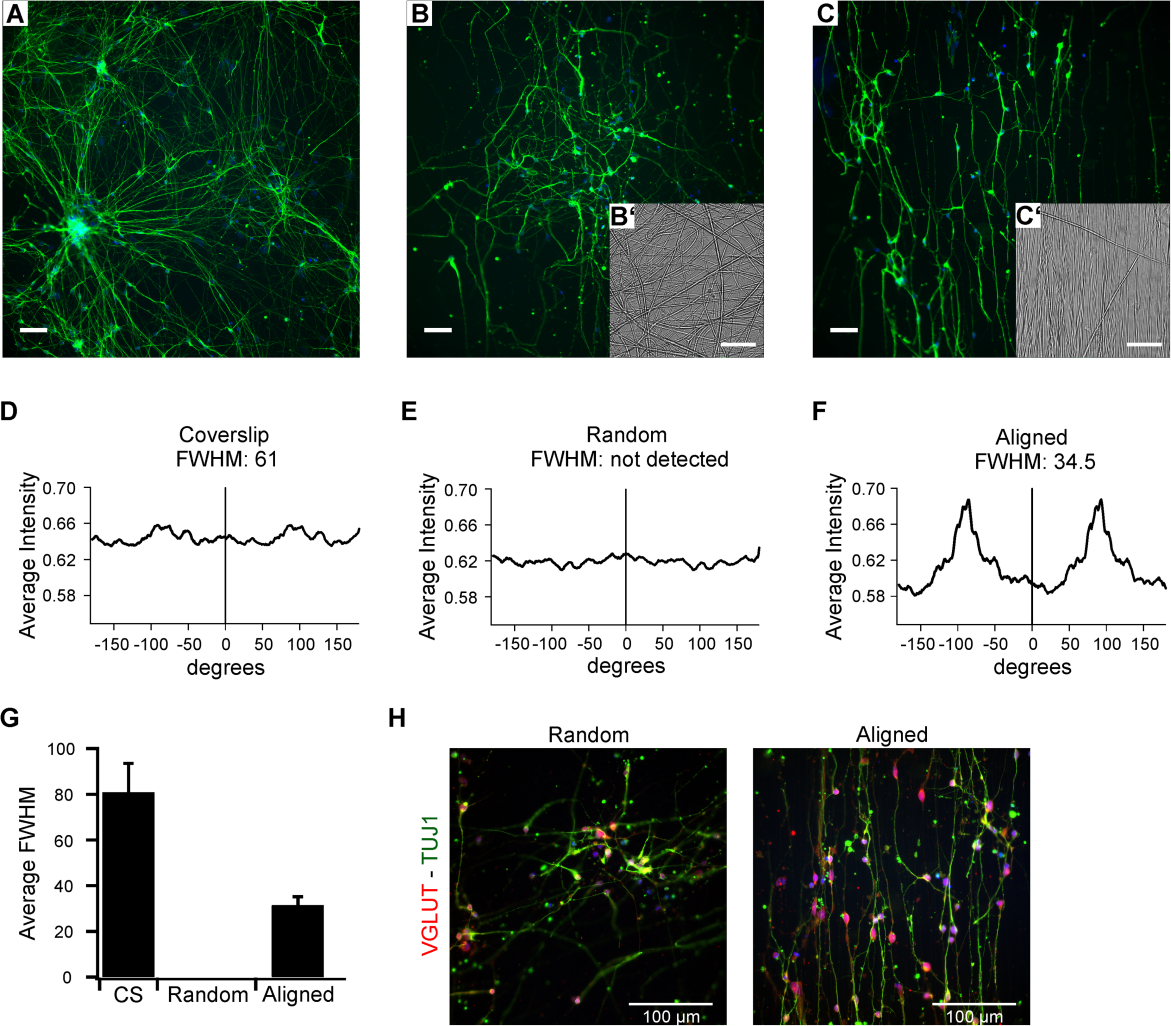
Neurite alignment on nanofiber mats *in vitro*. (A–C) Representative images of TUJ1 stained terminally differentiated H7 hESC derived neurons (green) on PS coverslips (A) and unaligned (B) and aligned (C) nanofiber mats. Insets in B’ and C’ show light microscopic images of unaligned and aligned nanofiber mats. (D–F) Plot of image intensity as a function of angle with corresponding FWHM values after Fourier transformation of fluorescence images of A, B and C illustrating the extent of alignment. A low FWHM indicates high alignment. (G) Quantification of FWHM values of coverslips (n = 8) and unaligned (n = 7) and aligned (n = 6) fibers terminally differentiated for 2–6 weeks. For random samples, only one out of 7 samples allowed FWHM calculation. (H) Immunohistochemistry for TUJ1 (green) and VGLUT1 (red) shows that neurons maintain a glutamatergic fate on fiber mats. Scale bars represent 100 μm in A, B, C and H and 50 μm in B’ and C’.

Before introducing NPCs to scaffolds for implantation, the capability of nanofibers to guide neurite outgrowth was examined *in vitro*. NPCs were seeded either on polystyrene (PS) plastic coverslips or unaligned or aligned polycaprolactone (PCL) nanofiber mats, terminally differentiated and stained for the neuronal marker TUJ1 (Fig 2A–2C). Alignment was verified by peak analysis of Fourier transformations of TUJ1-labeled images, where periodic patterns in the spatial images emerge as peaks at particular frequencies in the spectral domain. The average magnitude of the fast Fourier transform was plotted against polar angle to obtain full-width, half-max (FWHM) values, where a low value indicates a narrow peak and a high degree of alignment (Fig 2D–2F). Coverslips revealed broad peaks with high FWHM values (n = 8). The appearance of some alignment in these images reflected the tendency for neurites to fasciculate and project between small clusters of cell bodies. Cells grown on random nanofiber mats extended neurites along the randomly oriented fibers, rendering featureless FFTs and an inability to determine FWHM for these samples (n = 7). In contrast, neurons grown on aligned fibers (n = 6) produced narrow peaks in the FFT analysis and low FWHM values, indicating a high degree of alignment (Fig 2G). Hence, nanofiber orientation effectively guided the neurite outgrowth on aligned mats. Since nanofibers could potentially also affect phenotype characteristics, neurons grown on unaligned and aligned fiber mats were stained for VGLUT1 (Fig 2H). Glutamatergic neurotransmitter phenotype was maintained in both conditions.

**Fig 3:**
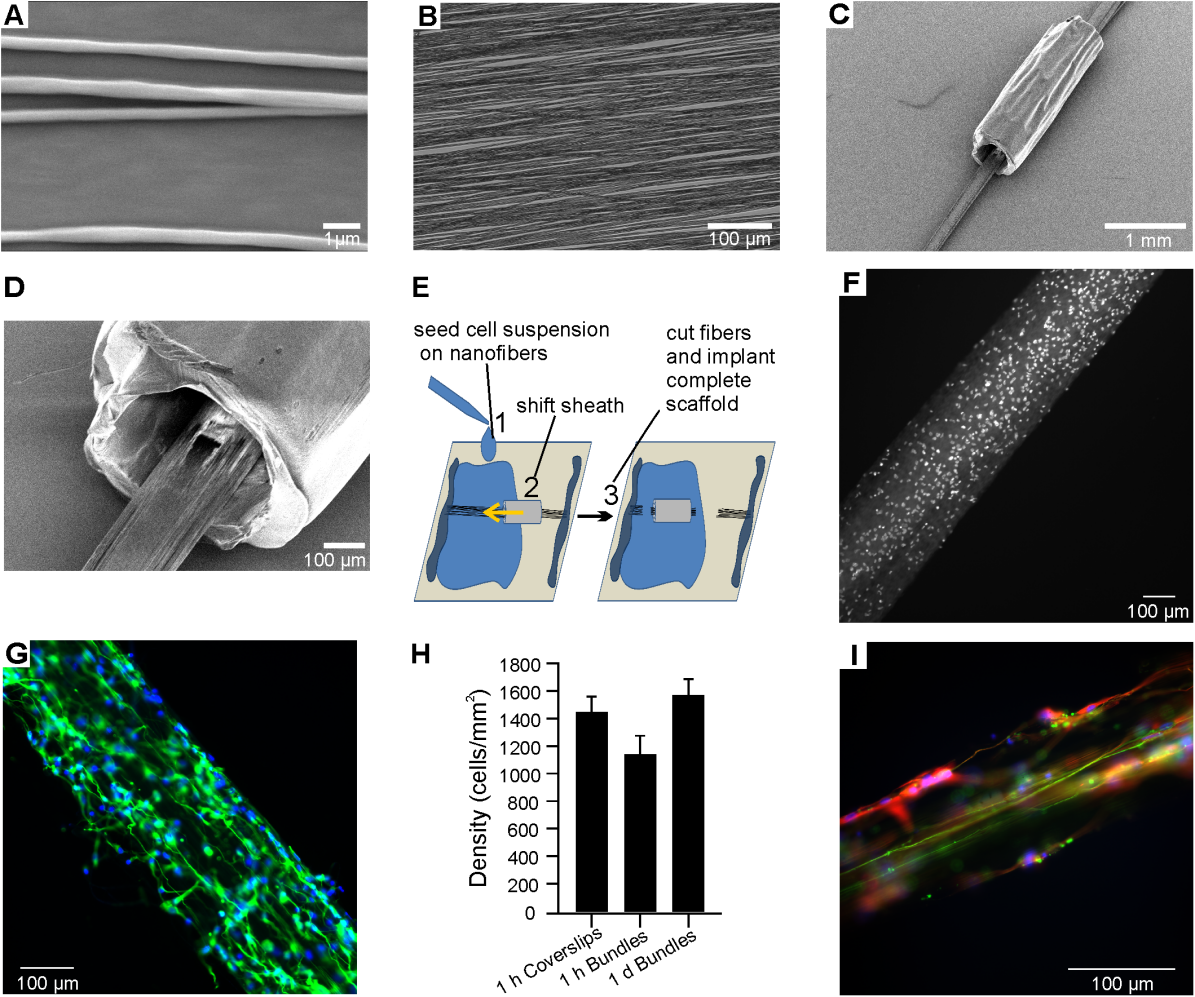
Nanofibrous scaffold assembly, NPC seeding and pre-implantation adhesion. (A and B) High resolution images of single (A, SEM image) and assorted (B, light microscopic image) PLLA:PCL nanofibers. (C and D) SEM images of assembled scaffolds consisting of nanofiber bundle and PCL sheath. (E) Illustration of procedures for seeding NPCs on nanofibers for implantation. A long nanofiber bundle with sheath was attached to a coverslip. (1) Concentrated cell suspension (10,000 cells/μl) was deposited on a nanofiber portion outside the sheath and the cells allowed to settle. (2) The sheath was shifted and positioned over the nanofiber area with attached cells. (3) The excess length of nanofibers was cut, releasing the scaffold for implantation. (F) Nuclear Hoechst stain of NPCs one hour after implantation. (G) TUJ1 stain (green, counterstained with Hoechst) shows fiber extension and alignment on Nanofiber bundles as early as 24h after seeding. (H) Quantification of Hoechst stain based cell counts on coverslips and bundles of parallel cultures. Error bars represent standard error of the mean. (I) Neurite extension aligned with nanofiber orientation 6 weeks after seeding (green: TUJ1, red: Nestin, blue: Hoechst). Scale bars represent 1 μm in A, 100 μm in B, D, F, G and I and 1 mm in C.

To provide guidance for NPC neurites upon implantation in the guinea pig IAM, we designed nanofiber scaffolds consisting of bundled PLLA:PCL nanofibers and a PCL sheath. Representative images of single nanofibers and aligned fiber mats are shown in Fig 3A and 3B (fiber diameter < 1 μm). Fiber bundles vacuumed through the PCL sheath maintained their alignment (Fig 3C). These bundles occupied 15–25% of the sheath cross-sectional area (Fig 3D). Fraying of the fibers at the opening of the sheath and degradation of the alignment prevented formation of larger bundles within the sheath. As the surface of the nanofibers is not readily accessible inside the sheath, we developed a seeding approach that involved depositing NPCs on an exposed bundle portion and subsequently enclosing the area in the sheath after the cells were allowed to settle and attach to the nanofibers, as illustrated in Fig 3E. A suspension of NPCs was added to the coverslip, bathing an exposed region of the fiber bundle pre-drawn through the polymer sheath (Fig 3E, Step 1). After 1 h in culture, the NPCs settled and adhered to the surface of the fiber bundle, allowing us to shift the sheath over the seeded expanse (Fig 3E, Step 2). The sheath containing the NPC-seeded fibers was cut free (Fig 3E, Step 3) and continued in culture for 24 h before implantation or for up to 6 weeks for *in vitro* assays. Coverslips and unused portions of the seeded nanofiber bundles were routinely stained for Nestin and TUJ1 to assess the quality of the NPC cultures and consistency of the density of attached cells. At the time of implantation, 78.2 ± 2.7% and 30.0 ± 4.8% (N = 10) of the cells were Nestin-positive and TUJ1-positive, respectively. A small percentage of Nestin- and TUJ1-negative cells (<5%) were identified; these appeared to be contaminating flat cells likely transferred with NPCs during rosette selection. Nuclear staining and immunocytochemistry for TUJ1 confirmed presence of NPCs on bundles 1 h post seeding (Fig 3F) and outgrowth of neurites after 24 h (Fig 3G) just before implantation. Thus, neurite outgrowth started prior to implantation. To assess the efficiency of NPC attachment, we compared cell density on bundles 1 h and 24 h after seeding to the cell density on the coverslip adjacent to the exposed fiber bundle (Fig 3H; coverslips n = 24, 1h bundles n = 25, 24h bundles n = 5). Cell density was unchanged over time (one-way ANOVA, *p* > 0.05). Scaffolds cultured in TD media for 6 weeks showed continued neurite outgrowth and alignment (Fig 3I). Even after long-term culture, some Nestin-positive cells persisted alongside TUJ1-positive neurites, indicating that some NPCs remain in an immature state *in vitro*. To facilitate detection and unequivocal identification of the hESC derived NPCs once implanted into the guinea pig, we used a modified stem cell line (H9-GFP) heterologously expressing the green fluorescent protein variant hrGFP for implantation. qPCR and immunocytochemistry confirmed the successful differentiation of these cells to a glutamatergic phenotype comparable with H7 cells (S1 Fig).

**Fig 4:**
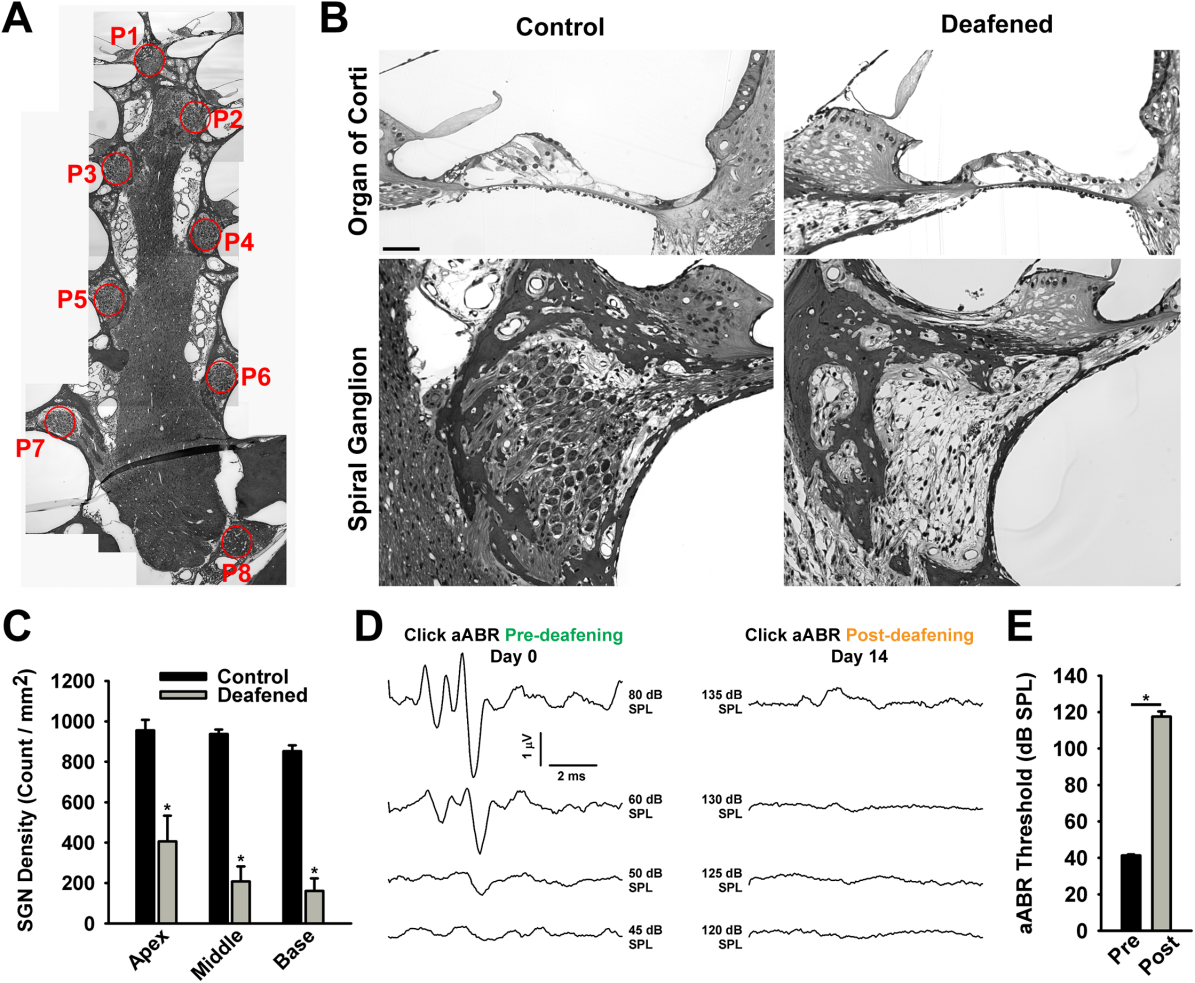
Efficacy of ouabain deafening in guinea pigs pre-implantation. **A.** Image collage of a mid-modiolar plastic section with positions of Rosenthal’s canal cross sections labelled from P1 to P8. (B) Representative images of spiral ganglia and the organ of Corti in untreated control (left) and ouabain deafened specimen (right, scale bar = 50 μm). SGN somata can be seen as dark round circles within the bony structure of the Rosenthal’s canal in control, but are depleted following ouabain treatment. Accompanying the loss of somata is a loss of fiber structures, which in control subjects project to organ of Corti and modiolus. (C) Quantification of SGN depletion along the cochlear axis from apex to base (control: n = 4, deafened: n = 5, * *p* < 0.001 for each region). (D) Click aABR waveforms elicited at increasing sound pressure levels (SPL) in a guinea pig pre- and post-deafening. (E) Quantification of aABR thresholds shows significant threshold elevation by ouabain treatment (* *p* = 0.01, n = 19), confirming efficient deafening at a physiological level. The pre-implantation deafening data include animals from sham, 1 and 2 month cell-free scaffold and 1 month NPC-scaffold groups. Error bars represent standard error of the mean.

The target group for nanofibrous implants is patients with severe hearing loss due to auditory nerve damage. To model nerve damage in guinea pigs and investigate the potential of our implants to improve nerve regeneration and restore hearing, we sought to pharmacologically deplete auditory neurons before implantation. The glycoside ouabain, a Na+/K+ ATPase inhibitor [70], has been shown to selectively deplete SGNs in gerbil [71] and mouse [72]. However, Hamada and Kimura [73] reported preferential targeting of hair cells in guinea pig, and experiments in rats [74] showed SGN and hair cell loss in a concentration dependent manner. We hypothesized that anatomical differences in these species contributed to the differences in response to ouabain. Therefore, to establish a guinea pig deafness model with SGN loss, we reexamined ouabain as a deafening agent combining round-window application with direct injection into the cochlear duct. Neurotoxicity was accessed by anatomical observation of SGN survival and recording of click-evoked aABRs. For analysis of SGN survival, mid-modiolar plastic sections were prepared from ouabain-treated and control animals. Fig 4A shows a collage of a mid-modiolar section through a control ear, illustrating the apical-to-basal cross-sections of Rosenthal’s canal (P1-to-P8) containing the spiral ganglion cell somata. Fig 4B shows representative images of the Rosenthal’s canal as well as the organ of Corti in control and treated ears. Deafened ears showed a severe loss of SGN somata, while organ of Corti damage was found to be variable. Quantification of Rosenthal’s canal cross-sections grouped into apex, middle and base of the cochlea revealed that ouabain treatment affected each area compared to controls, but neuronal depletion was less profound in the apex (Fig 4C, control n = 4, deafened n = 5, two-way ANOVA with Tukey post-hoc pairwise comparisons, *p* < 0.001 for each region). Overall, the treatment resulted in a loss of 72% of SGNs. Auditory neuron depletion was accompanied by physiological hearing impairment, evident from flattened aABR waveforms (Fig 4D) and a significant aABR threshold increase of nearly 80 dB (Fig 4E, n = 19, Student’s t-test, *p* < 0.001). Taken together, SGN counts and hearing thresholds demonstrate ouabain treatment effectively damaged guinea pig primary auditory neurons.

**Fig 5:**
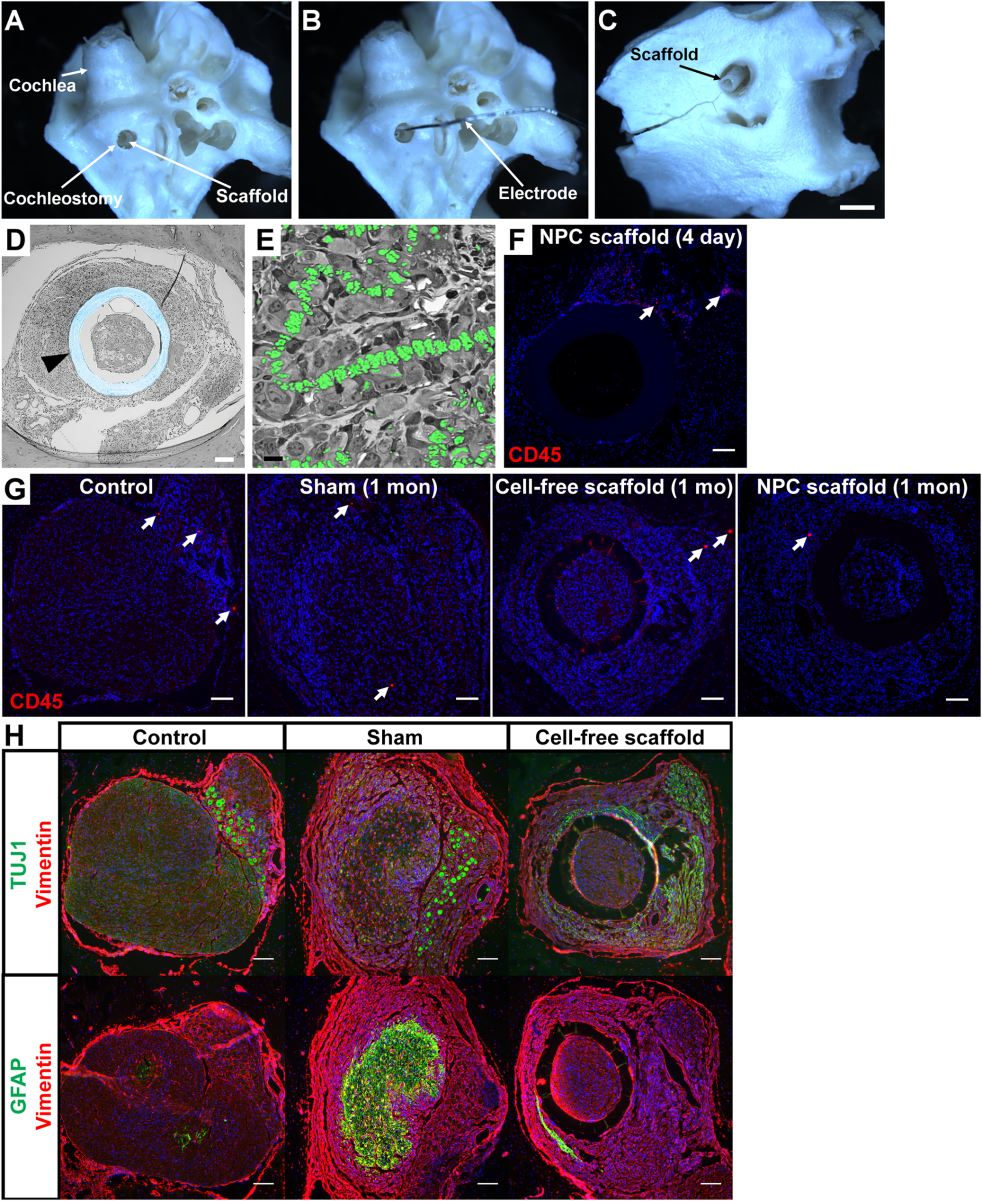
Surgical approach and histological assessment of placement and tissue response. (A–C) Guinea pig temporal bone showing the access to and the scaffold placement in the IAM. (A) The scaffold was advanced into the IAM through a cochleostomy in the base of the cochlea. (B) Positioning of the electrode co-implanted for eABR recordings. (C) View on the positioned scaffold from the brain side. (D and E) Plastic cross-sections of the IAM with implanted cell-free scaffold 15 days post-implantation. (D) Cross section showing the IAM with its bony wall and the scaffold (arrowhead; sheath shaded in blue) embedded in host tissue. Host tissue was found to infiltrate the full length of the scaffold. (E) Higher magnification of the scaffold’s interior with host tissue embedding the nanofibers (shaded in green), which adopt a chain-like arrangement. (F) IAM immunohistochemistry for the hematopoietic lineage marker CD45 shows few immune cells within the IAM 4 days after implantation with an NPC-seeded scaffold. (G) Additional samples 1 month post-implantation were examined for CD45 labeling, including sham, cell-free scaffold, and NPC scaffold animal groups, as well as untreated controls. Few immune-positive cells were identified in these sections indicating that the surgery, scaffolds, and NPCs did not trigger a significant immune response. (H) Immunohistochemistry for TUJ1 (neurons), GFAP (glia) and vimentin (Schwann cells/fibroblasts) in control, sham and cell-free scaffold (1-month post implant) shows involvement of vimentin and GFAP positive cells in tissue repair. Scale bars represent 1 mm in A—C, 100 μm in D—H.

To access the guinea pig IAM for scaffold implantation, we developed a surgical approach utilizing a cochleostomy at the basal turn inferior to the round window niche to expose the IAM opening. An inner ear explant illustrates the location of the defect as well as the positioning of the scaffold (Fig 5A) and the electrode co-implanted for eABR measurements (Fig 5B) from the cochlear side. The scaffold can also be seen exiting the IAM from the brain side (Fig 5C). *In vivo* positioning of the scaffolds and tissue response to implantation was evaluated by preparation of plastic sections and immunohistochemistry of cryosections. Plastic sections of cell-free scaffolds confirmed successful and stable placement in the IAM (Fig 5D) and revealed that the space between the sheath and fibers as well as between fibers was invaded by host cells (Fig 5D and 5E). Fibers were typically found to exhibit a chain-like arrangement in cross section (Fig 5E) as a consequence of forming the fiber bundle by vacuuming a mat of fibers. While examination of plastic sections indicated a lack of a notable inflammatory response, we further tested for an immune response by staining for the hematopoietic lineage marker CD45. As shown by a representative image in Fig 5F, few CD45-positive cells were found 4 days post-implantation. To determine whether the number of CD45-positive cells changed over time and whether the presence of a few positive cells at day 4 was normal for control ears, we examined additional samples from all treatment groups, including control ears as well as 1 month post-implant samples from sham, cell-free scaffold, and NPC scaffold groups (Fig 5G). As in the short-term implant, a small number of sections revealed few CD45-positive cells. Notably, surgically manipulated animals showed no increase in immune cells over control tissue. Guinea pig blood smears were used as positive control (data not shown). Similar results were found using additional markers for immune response (CD4, CD11b, and Macrophage L1; S2 Fig). To identify the nature of the tissue response, we stained control, sham and cell-free scaffold sections for the neuronal marker TUJ1, the glial marker GFAP and the Schwann cell and fibroblast marker vimentin (Fig 5H). In untreated control animals, cross sections of thin neuronal fibers are stained by TUJ1 and interspersed Schwann cells by vimentin, while there is only a minor presence of GFAP-positive glial cells. At the position of the Scarpa’s ganglion of the vestibular nerve, neuronal somata are enfolded by Schwann cells. Analysis of sham and cell-free scaffold preparations indicates that both GFAP- and vimentin-positive cells are involved in the guinea pig tissue response. Examination of scaffold implants reveals a general presence of vimentin-positive cells in and outside the sheath, while GFAP staining is found to be increased but excluded from the scaffold interior. These findings suggest that the major tissue response represents tissue repair, and that host scaffold invasion is carried by vimentin-positive cells, presumably representing Schwann cells.

**Fig 6:**
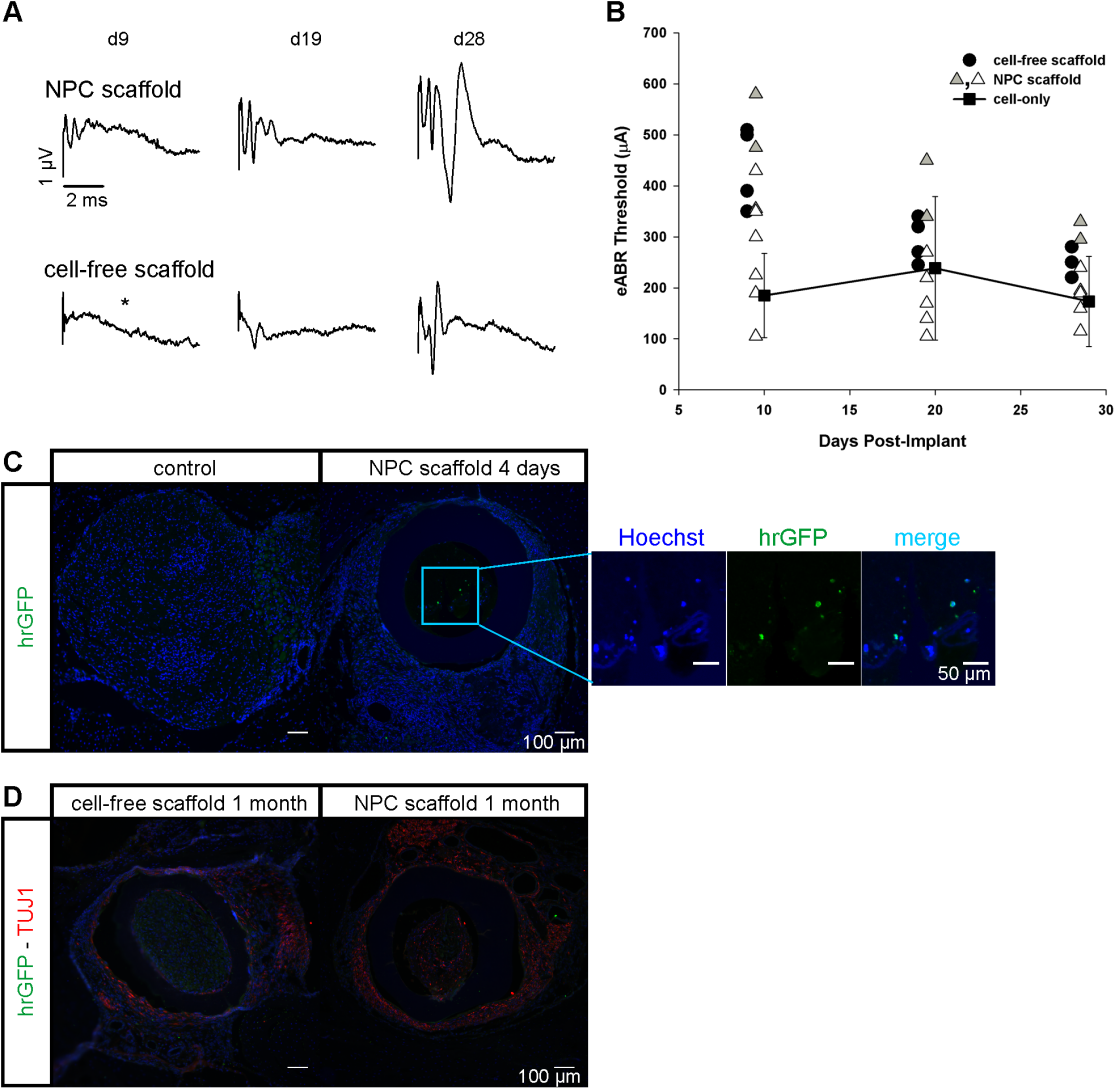
Post-implant physiology and histological assessment of H9-GFP derived NPC survival. (A) Representative examples of in vivo threshold eABR waveforms of cell-free and NPC scaffolds at post implantation days indicated. In both groups, waveforms are flattened after deafening and implantation. Profiles of distinct waves partially recover over the one month observation period. All responses were evoked with a 300 μA stimulus, except for the day-9 time point of the cell-free scaffold subject (indicated by *, 300 μA was below threshold and thus a 370 μA response is shown for illustration). (B) Course of post implantation eABR thresholds for individual animals implanted with cell-free scaffolds (n = 4) and NPC scaffolds (n = 9) is shown alongside the mean thresholds for NPC-only injections (n = 3). Gray triangle symbols indicate two animals implanted with dye-labeled NPC-scaffolds; the elevated thresholds in these animals may indicate a negative impact from the FluoroRuby dye on NPC health. Data points are slightly shifted in time for clarity. (C) hrGFP immunohistochemistry for the detection of H9-GFP derived NPCs in sections of control and implanted specimen collected 4 days post implantation. The excerpt shows a magnification of the interior of the scaffold with hrGFP expression in two Hoechst-positive cells (blue channel enhanced for illustration). (D) There was no hrGFP signal detected in 1 month implantations. TUJ1 stain shows interspersed fibers both in cell-free and NPC-seeded conditions. Scale bars represent 100 μm in C and D and 50 μm in the excerpt of C.

The ability of the combination of NPCs and nanofibers to improve physiological nerve function was evaluated by comparing eABR thresholds of NPC scaffolds to cell-free scaffolds and NPC-only injections 9, 19 and 28–30 days after implantation. Representative examples of the respective eABR waveforms for NPC and cell-free scaffolds are given in Fig 6A. Amplitudes increased over time, indicating greater neural response whether for cell-free and NPC scaffolds suggesting some recovery in both experimental conditions. eABR thresholds for individual scaffold-implanted animals, with (n = 9) and without NPCs (n = 4), are shown in Fig 6B alongside the average thresholds for additional animals implanted with NPCs only (n = 3). A two-way ANOVA was used to examine statistically reliable changes in eABR thresholds over time and between scaffold-implanted groups. Thresholds decreased significantly for this dataset over time (*p* = 0.004), but there was no reliable effect between scaffolds with or without NPCs (*p* = 0.100), indicating a partial functional recovery independent of the presence of NPCs. Markedly, NPC-only injections exhibited a less severe initial threshold elevation and a lack of recovery over the observation period (one-way ANOVA over time, *p* = 0.902), emphasizing the initial damaging effects of scaffold implantation in the other treatment groups. Two animals implanted with NPC-seeded scaffolds were treated with FluoroRuby prior to implantation in an effort to assist in cell tracing *in vivo*. While the labeling approach did not improve detection of implanted cells, we noted that the eABR thresholds for these two animals were highest among the NPC-scaffold implant group (Fig 6B; gray triangle symbols). While neurons generally tolerate FluoroRuby staining well, the pre-treatment of NPCs with FluoroRuby or the extra day in culture prior to implantation may have led to poorer outcomes. If these two animals are eliminated from the dataset, the two-way ANOVA indicates a reliable effect between scaffolds with or without NPCs (*p* < 0.001), with post-hoc pairwise comparisons showing a statistically significant effect at day 9 only (*p* = 0.027).

In order to test if the poor long-term outcome of NPC scaffolds was due to a lack of cell survival or differentiation to an undesired fate, we performed immunohistochemistry on cryosections of the specimen. To establish whether we could detect the exogenous cells post-implant, we examined serial sections through two animals only 4 days post-implantation with the NPC scaffolds. Using immunocytochemistry targeting the hrGFP expressed by the NPCs, we could detect the presence of implanted cells within the guinea pig IAM (Fig 6C). Notably, the number of hrGFP-positive cells detected in these 4-day implants was low; approximately 80 cells per scaffold could be reliably counted (n = 2, examination of 153 sections, data not shown). Comparison with cell-free scaffolds indicated a false-positive detection rate of up to 12% (n = 1, 26 sections). Even so, this cell density, which is roughly 10% of the seed density, is a conservative estimate because scaffolds were frequently lost during staining and some cells could have migrated out of the scaffold interior. Indeed, we often found hrGFP cells were detached from the nanofiber bundle and positioned along the wall of the sheath or freely within the interior. After 1 month, no hrGFP-positive cells could be detected (Fig 6D). In addition, we tested for the presence of neuronal fibers. TUJ1 staining showed a low number of neurites and neuronal cells within the scaffold in both cell-seeded and cell-free conditions (Fig 6D and data not shown).

## Discussion

Efforts to regenerate the auditory nerve thus far have concentrated on supporting remaining spiral ganglion fibers and finding or alternatively recreating an equivalent replacement neuron. However, strategies to ensure the proper integration into the auditory circuit within its complex anatomy are largely lacking at this stage. Most studies published to date have relied on implanted neurons to autonomously integrate themselves into an existing or damaged system [25,28,30–33,75]. These attempts have served as important first proof of principle, and in some cases indicated that graft cells can indeed achieve physiological improvement [23,30]. However, it also became clear that unguided wandering, branching to alternate paths, cross-talk, and innervation of off target cells by implanted neurons are significant hurdles for an efficient functional integration. Stem cell differentiation protocols that produce cells closely resembling SGNs may confer all the information necessary for successful integration in a developmental context, but further tissue engineering will likely be required to guide integration in the adult. With respect to successful treatment of auditory neuropathy in a variable patient population, it is crucial to implement strategies for a reliable and efficient nerve replacement.

Here, we designed novel nanofibrous scaffolds that were fit to the guinea pig IAM. Implanting scaffolds together with hESC-derived NPCs addresses two goals of auditory nerve replacement: replacing SGNs depleted in auditory neuropathy and guiding those cells to integrate into the auditory circuit. In this context, guidance encompasses both production of a bipolar neuronal morphology and the fasciculation of neurites throughout the length of the IAM, such that neurites can enter their target area of the cochlea and the cochlear nucleus at both ends of the scaffold.

Using a stepwise reprogramming strategy involving BMP inhibition and SHH/RA signaling, we derived NPCs that highly expressed proneural (*ASCL1*) and regionalization (*PAX6*) transcription factors consistent with forebrain progenitors [76]. However, we also found upregulation of other proneural (*NEUROG1*) and regionalization (*PAX2/8*, *POU4F1*, and *GATA3*) factors consistent with sensory placodes [77]. While the degree of overlapping expression at the level of individual neurons remains uncertain, the upregulation of multiple regional factors suggests some heterogeneity or generalization in fate specification that ultimately converged to produce a glutamatergic phenotype. Moreover, these neurons were capable of extending processes along aligned nanofibers, in accordance with previous reports of neurite alignment [38,41–43]. Alignment was observed both on mats and bundles of nanofibers in culture. However, limitations in identifying implanted cells and the 3-dimensional nature of implanted scaffolds precluded an analysis of alignment and integration of NPCs *in vivo*.

Cell survival appeared to be a critical barrier to success in our approach. Based on the average cell density on fiber bundles (1,500 cells/mm^2^) and the average surface dimensions of the fiber bundle (1.9 mm x 0.7 mm), about 2,000 cells were implanted per scaffold. According to our assessments of short-term implantations, about 5–10% of the cells survived 4 days after surgery. This estimate is conservative since some sections were lost or obscured. However, a loss of >90% of implanted neural progenitors is not uncommon [78,79], and cell death is a common problem in stem cell implantation studies in the inner ear [51]. Our limited ability to identify implanted NPCs in short- and long-term implantation could reflect cell death as well as cell migration and epigenetic effects on reporter gene expression. Migration of the implanted cells is one possible contributor to reduced cell counts. In fact, many cells found within the sheath were not tightly associated with the nanofibers, indicating a lack of cell adhesion, an observation also made in long-term cultures *in vitro* (data not shown). While it is possible, then, that the cells dislodged from the nanofibers and migrated out of the scaffold, we did not observe large numbers of surviving cells in cochlear sections or brainstem sections. Alternatively, the reduction in GFP-positive cells over time could reflect epigenetic downregulation of GFP. Silencing of constitutive reporters is common in xenografts [80]. However, since the number of Hoechst-positive cells was already extremely low after 4 days, the absence of cells after one month was likely due to cell death rather than confounded loss of the GFP reporter or substantial migration out of the graft. Taken together, we conclude that too few NPCs were implanted in our design and that there was significant attrition of the NPCs over time. Methods to improve cell survival and adhesion to the fiber bundle are required. Moreover, more sophisticated scaffold designs and seeding methods are required to facilitate implanting a larger number of NPCs so that some attrition does not ultimately lead to such a low number of surviving cells.

Optimization of future nerve regeneration studies will also have to address the characteristics of the implanted cells. In this study, we focused on scaffold design and chose to implant NPCs that share a selected number of characteristics with SGNs. Selection of a limited number of common features has been applied in previous stem cell implantations into the ear [29]. Moreover, it has even been argued that protocols tailored to a different, but similar neuron type could provide for a suitable SGN replacement [24]. Even so, no study so far has addressed the possibility that phenotypic characteristics may be modified by the local environment. Consequently, it remains unknown how much molecular and structural similarity is required in the *ex vivo* differentiation models to sufficiently model SGNs for replacement. Moreover, it is unknown how this will depend on subject age, brainstem plasticity, and the specific mode of nerve damage.

Hence, proper evaluation of nerve repair also depends on the neuropathy model. Here, we pharmacologically induced nerve damage by applying ouabain to the round window to enable assessment of implant dependent tissue responses and recovery of eABR thresholds. While studies in gerbil and mouse suggested SGN specific damage [71,72], studies in rats and guinea pig reported graded responses [74] or conflicting results [73,81] when examining cell target specificity with respect to sensory hair cells. In this study, local ouabain application resulted in preferential SGN loss, but did not entirely spare hair cells. Improvement of agent deposition may provide for a more SGN selective deafness model that will allow the study of hearing threshold regeneration after SGN replacement in guinea pigs.

One of the strategies pursued for auditory nerve regeneration is recruitment or modification of Schwann cells, underscoring their importance for neuron support [19]. Immunohistochemistry for the Schwann cell marker vimentin [82] performed in this study suggests abundance of Schwann cells in the IAM, which readily enter the scaffold. These findings imply there is no need for Schwann cell recruitment with our approach. However, future experiments with modified implants will have to further examine cell-cell interactions of Schwann cells with nanofibers and NPCs. Importantly, we did not observe evidence for an inflammatory response of the guinea pig tissue upon scaffold implantation which could create a hostile environment for neuronal survival.

To our knowledge, there is thus far one other study utilizing nanofiber support of neuronal cells in the auditory system *in vivo*. Jiao et al. [27] injected human forebrain neural precursors embedded in nanofiber gels constituted of self-assembling peptide amphiphile (PA) molecules that were linked with the extracellular matrix protein laminin epitope isoleucine-lysine-valine-alanine-valine (IKVAV) into deafened rat auditory nerve trunks by the IAM. Unfortunately, there was no indication of neurite alignment, and cells were found to migrate to the auditory brain stem. Our observations from short-term implants indicated poor long-term adherence of GFP-positive cells on the nanofiber substrates but did not show large-scale migration toward the brainstem. Future studies will have to address the utility of nanofiber gels in the auditory system. Valuable insights will also be gained from comparison to other sites of injury in the nervous system.

In spite of implant cell death, we observed a partial recovery of auditory thresholds after scaffold implantation. Observations after cochlear implantations in the guinea pig suggest that the surgical procedure alone can induce damage to the peripheral auditory circuit that subsequently largely recovers [83]. In our study, recovery of eABRs putatively represents combined recovery from deafening and scaffold implantation. Apart from SGNs, procedures most likely also affected hair cells, vestibular nerve, and efferent fibers. Physiological recovery and the absence of an inflammatory response are encouraging findings that suggest the presence of scaffolds is permissive to post-surgery recovery. Moreover, variable observations of fiber growth through the scaffolds show that recovery includes the passage of neurites and supports the idea that modification of nanofibers can eventually provide a favorable environment for nerve repair.

Several strategies could be employed to improve integration and functional recovery. The primary limitation in the *in vivo* aspect of the study is cell number, including the low density of cells originally seeded on the fiber bundles and the poor long-term survival of implanted cells. One option to potentially improve outcomes is implantation of terminally differentiated, mature neurons rather than NPCs. Failure to differentiate *in vivo* could have contributed to cell loss post-implantation, but concerns about mechanical manipulation of fully differentiated neurons on nanofiber scaffolds led us to focus on implantation of NPCs. Alternatively, to increase the number of cells surviving long-term, we recommend the addition of neurotrophic support [27]. Neurotrophic support could be delivered by hydrogel impregnated within the scaffold or within the nanofibers themselves [84]. In our design, nanofibers were drawn into the scaffold sheath by vacuum. A limited number of fibers could be incorporated using this method trading fiber density for preservation of alignment. A larger sheath in a larger animal model would enable new designs, such as rolled fiber mats that could fill the interior of the sheath and further facilitate increased cell density. And finally, surface chemistry can be used to create favorable conditions to optimize cell adherence and promote survival [36,37,85–88]. Our study represents the first attempt at guiding neuroprogenitor differentiation and growth on an implantable scaffold. A virtually unlimited number of modifications can now be made to enhance scaffold design and functional recovery.

## Conclusion

Nanofibers are promising tools to guide neurites of stem cell derived neurons within the boundaries and unique organization of the auditory system. The scaffolds and surgical procedures of this study present a first step to tackle the challenges posed by probing a nanofiber based approach to auditory nerve regeneration *in vivo*. Noteworthy, the human anatomy [89] provides both more space and an easier access to the IAM compared to the guinea pig model. After optimization in animal models, transition to humans might enable simplification of surgical procedures and reduce tissue damage in favor of auditory nerve regeneration. While there is still much need for further development of nanofibrous scaffolds, advances in biomaterials and bioprinting [90,91] promise ample opportunity to tailor eventual implants for application in humans to human specific anatomy and SGN characteristics [18].

## Supporting information

S1 Fig Neuronal differentiation of H9-GFP hESC. (A) Light microscopic image of an undifferentiated H9-GFP colony during maintenance culture. Cells were fixed and stained with Hoechst. Native hrGFP fluorescence is shown after fixation, unaided by antibody amplification. (B and C) Representative confocal images of differentiated H9-GFP cultures stained for the neuronal marker TUJ1, the glutamatergic phenotype marker VGLUT1 (B) and the synaptic vesicle protein synaptophysin (C). Native hrGFP fluorescence is shown in each image. (D) qPCR analysis for neuronal differentiation and otic placode associated markers shows upregulation of the genes of interest in H9-GFP cells, indicating the differentiation protocol effectively induces a glutamatergic neuronal fate. Error bars show standard error of the mean. H7 data are reproduced from Fig 1 for comparison. * indicates significance (*p* < 0.05). Scale bars represent 100 μm in A and B and 10 μm in C.
(TIF)

S2 Fig Inflammatory host-response post-implantation with NPC-seeded scaffolds. Representative IAM cryosections are shown for NPC-seeded animals stained with antibodies to macrophages and microglia, including the leukocyte common antigen CD45, the microglia/macrophage glycoprotein CD4, the leukocyte and microglial marker CD11b, and the L1 macrophage marker neural cell adhesion molecule L1 (L1cam/calprotectin). Images are representative of 2 to 3 animals and 10 to 15 sections throughout the IAM from each animal. Arrows point to immunolabeled cells associated with Hoechst-positive nuclei. No samples showed positive stain for CD11b.
(TIF)
