# Supplementary figures and images for: Nanofibrous scaffolds for the guidance of stem cell-derived neurons for auditory nerve regeneration
Source: PLoS One. 2017 Jul 3;12(7):e0180427. doi: 10.1371/journal.pone.0180427 (PMC5495534; doi:10.1371/journal.pone.0180427)

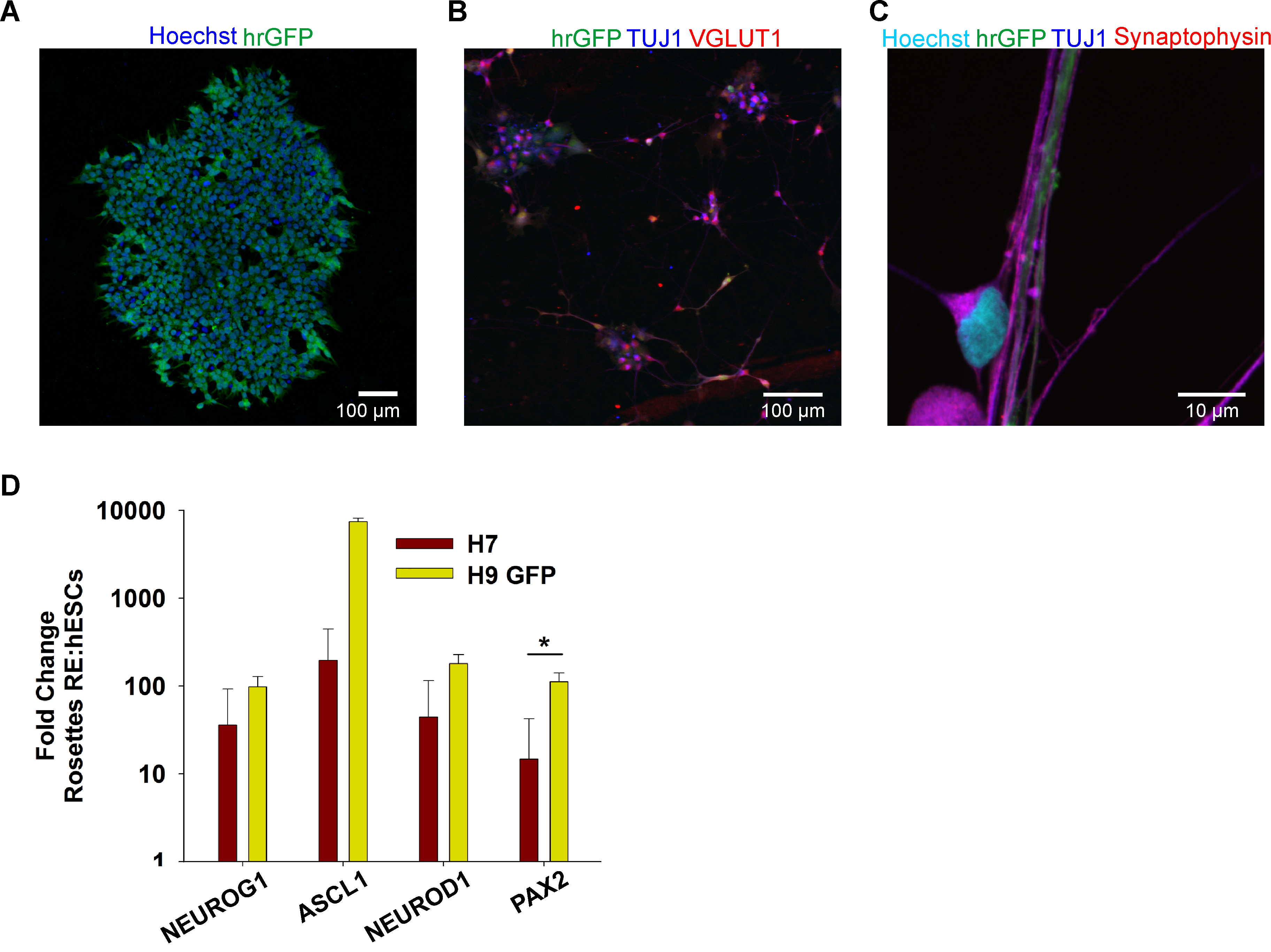

Supplement: S1 Fig — (A) Light microscopic image of an undifferentiated H9-GFP colony during maintenance culture. Cells were fixed and stained with Hoechst. Native hrGFP fluorescence is shown after fixation, unaided by antibody amplification. (B and C) Representative confocal images of differentiated H9-GFP cultures stained for the neuronal marker TUJ1, the glutamatergic phenotype marker VGLUT1 (B) and the synaptic vesicle protein synaptophysin (C). Native hrGFP fluorescence is shown in each image. (D) qPCR analysis for neuronal differentiation and otic placode associated markers shows upregulation of the genes of interest in H9-GFP cells, indicating the differentiation protocol effectively induces a glutamatergic neuronal fate. Error bars show standard error of the mean. H7 data are reproduced from Fig 1 for comparison. * indicates significance (p < 0.05). Scale bars represent 100 μm in A and B and 10 μm in C. (TIF) [file pone.0180427.s001.tif]

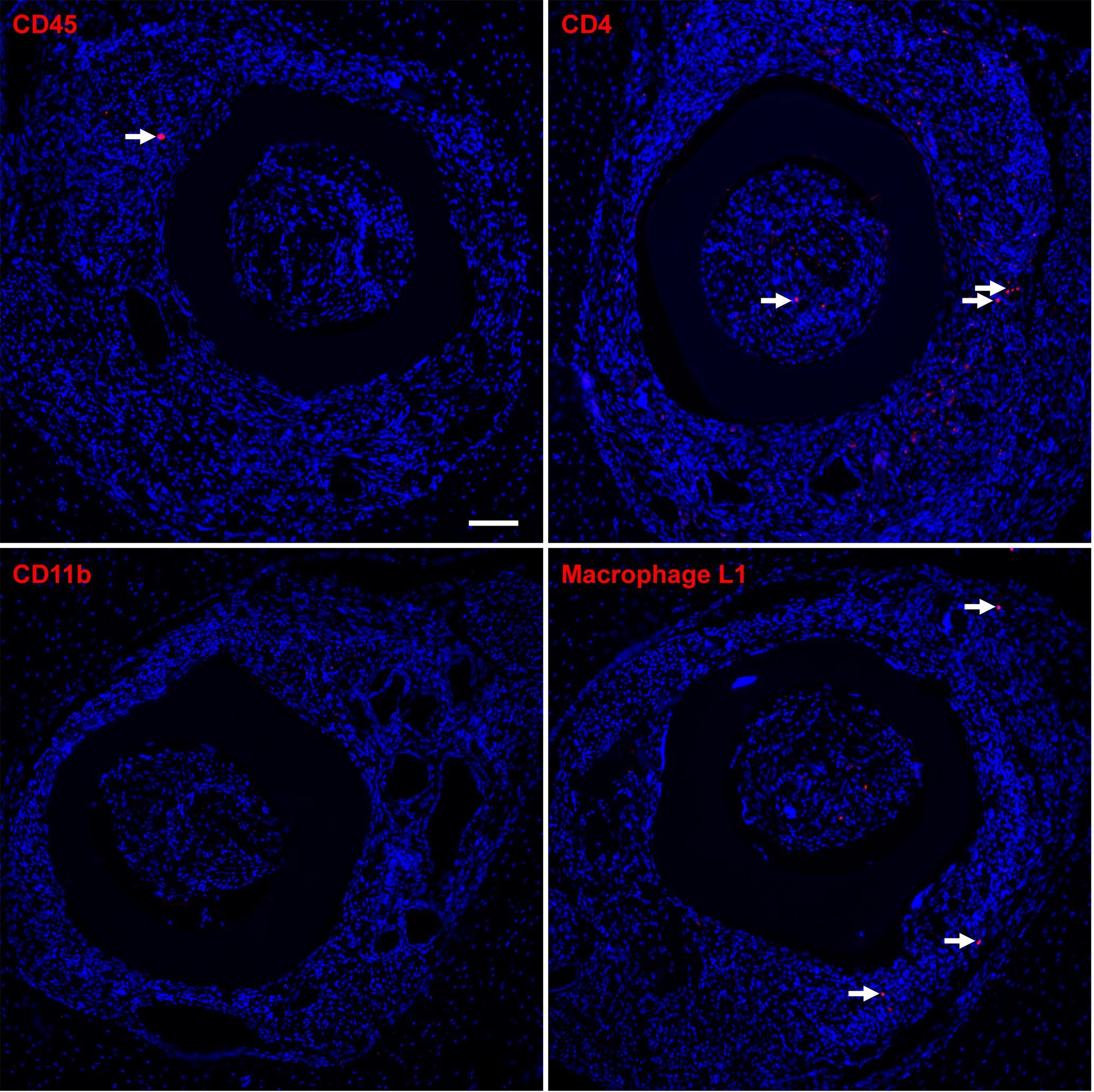

Supplement: S2 Fig — Representative IAM cryosections are shown for NPC-seeded animals stained with antibodies to macrophages and microglia, including the leukocyte common antigen CD45, the microglia/macrophage glycoprotein CD4, the leukocyte and microglial marker CD11b, and the L1 macrophage marker neural cell adhesion molecule L1 (L1cam/calprotectin). Images are representative of 2 to 3 animals and 10 to 15 sections throughout the IAM from each animal. Arrows point to immunolabeled cells associated with Hoechst-positive nuclei. No samples showed positive stain for CD11b. (TIF) [file pone.0180427.s002.tif]
